# Meta-fibrosis links positive energy balance and mitochondrial metabolism to insulin resistance

**DOI:** 10.12688/f1000research.11653.1

**Published:** 2017-09-27

**Authors:** Daniel S. Lark, David H. Wasserman

**Affiliations:** 1 Department of Molecular Physiology & Biophysics, Vanderbilt University School of Medicine, Nashville, TN, USA; 2 Vanderbilt Mouse Metabolic Phenotyping Center, Vanderbilt University School of Medicine, Nashville, TN, USA

**Keywords:** insulin resistance, metabolism, metabolic syndrome, inflammation

## Abstract

Obesity and insulin resistance often emerge from positive energy balance and generally are linked to low-grade inflammation. This low-grade inflammation has been called “meta-inflammation” because it is a consequence of the metabolic dysregulation that can accompany overnutrition. One means by which meta-inflammation is linked to insulin resistance is extracellular matrix expansion secondary to meta-inflammation, which we define here as “meta-fibrosis”. The significance of meta-fibrosis is that it reflects a situation in which the extracellular matrix functions as a multi-level integrator of local (for example, mitochondrial reactive oxygen species production) and systemic (for example, inflammation) inputs that couple to cellular processes creating insulin resistance. While adipose tissue extracellular matrix remodeling has received considerable attention, it is becoming increasingly apparent that liver and skeletal muscle extracellular matrix remodeling also contributes to insulin resistance. In this review, we address recent advances in our understanding of energy balance, mitochondrial energetics, meta-inflammation, and meta-fibrosis in the development of insulin resistance.

## Introduction

Advances in industrial and agricultural technology combined with lower rates of energy expenditure through physical activity have had the unintended consequence of creating a dramatic rise in the prevalence of obesity, insulin resistance (IR), hypertension, and dyslipidemia. These comorbidities are principal components of the metabolic syndrome as well as risk factors for type 2 diabetes mellitus and cardiovascular disease. The public health impact of these altered metabolic states is clear when considering that, in 2012, approximately 33% of United States citizens (over 100 million people) were projected to have at least one component of the metabolic syndrome ^1^.

**Figure 1.**
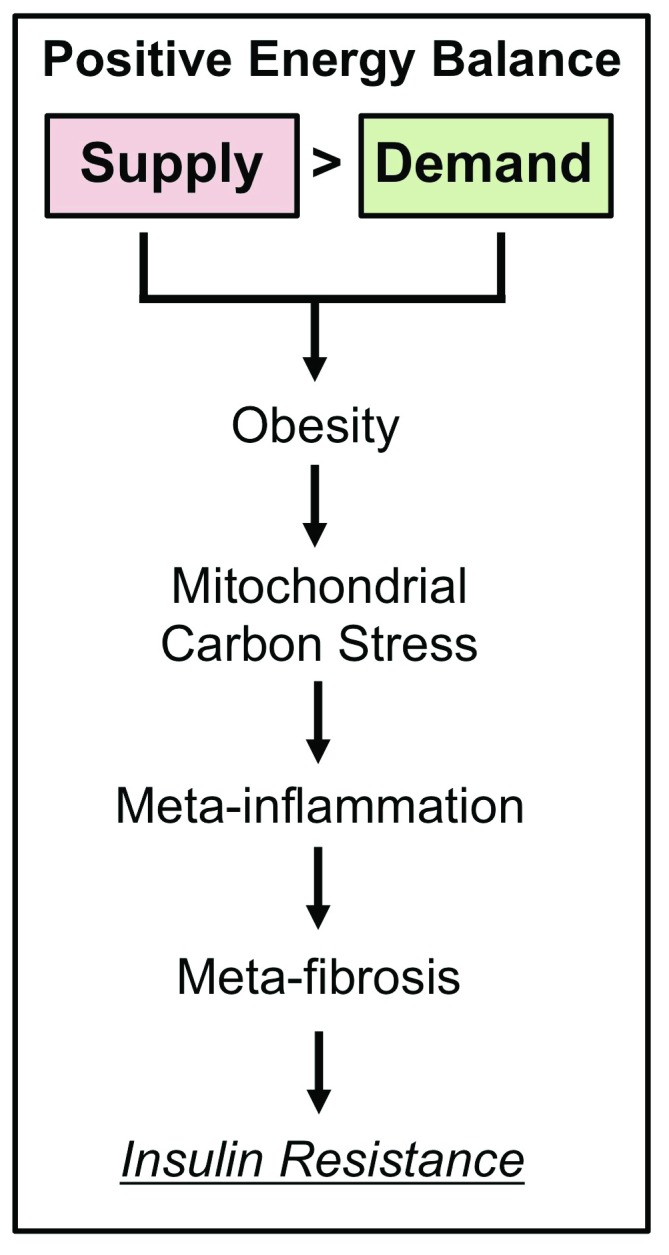
Positive energy balance promotes insulin resistance via metabolism-driven inflammation and fibrosis. Energy balance is defined as the difference between absorbed dietary macronutrients (Supply) and energy expenditure (Demand). Energy supply is determined by the quantity and composition of macronutrients consumed, whereas energy demand is determined by exercise, non-exercise activity thermogenesis, and resting metabolic rate. A net positive energy balance (Supply > Demand) leads to obesity and a cascade of events that includes mitochondrial carbon stress (that is, an oversupply of macronutrients to mitochondria). This metabolic stress on mitochondria can promote meta-inflammation and meta-fibrosis that ultimately contribute to cellular and systemic insulin resistance.

Positive energy balance at the whole-body level and altered oxidative metabolism at the cellular level are central to the development of IR. However, the conduit linking nutrient status and cellular energetics to pathophysiological states like IR is incompletely defined. In this commentary, we provide a framework for how mitochondrial energetics along with metabolically driven inflammation (meta-inflammation) and extracellular matrix (ECM) remodeling leading to fibrosis (meta-fibrosis) link overnutrition to IR ( Figure 1). As several recent discoveries suggest, there is a great deal to be learned regarding the etiology of IR by studying organ-level physiological events in the context of the extracellular milieu. The focus here will be on metabolism, molecular organization, and cell signaling in the pathogenesis of IR. The important roles of gene transcription and epigenetics in the development of IR are beyond the scope of this commentary. Readers are directed to recent reviews on these topics ^2, 3^.

## Energy balance and the metabolic syndrome

Energy balance is defined as the gastrointestinal absorption of dietary macronutrients minus whole-body energy expenditure. Human evolution has selected for traits that facilitate the efficient mobilization, metabolism, and storage of macronutrients. The biological significance of these adaptations lies in the need to store nutrients during times of nutrient excess and the ability to mobilize fuel in situations of nutrient deficiency. Nutrient storage is important for acute bouts of elevated energy expenditure or prolonged periods during which food is not readily available. Indeed, mechanisms for storing excess glucose (glycogen), lipids (triglyceride), and amino acids (protein) obtained from the diet are exquisitely sensitive. While these adaptations have been critical for survival and species propagation, people living in industrialized societies now have easy access to high-calorie foods and do not need to expend considerable energy to obtain their food. This has led to a sustained positive energy balance. Since this is a situation rarely encountered during the course of human evolution, the body is poorly equipped to adapt to dietary excess. As such, the chronic energy surplus incurred by overnutrition and sedentary behavior has become a persistent metabolic burden that leads to adipose tissue expansion and obesity in many individuals ^4^. Obesity, in turn, is central to the development of IR.

The evolutionarily conserved mechanisms that make survival possible during periods of famine also make humans refractory to weight loss. Resistance to weight loss and weight maintenance is recognized as a primary barrier to improving metabolic health ^5^. This is most clearly demonstrated when considering the effects of caloric restriction and physical activity on energy balance and body weight. In both obese and non-obese humans ^6– 8^, prolonged caloric restriction results in significant weight loss, but it is accompanied by reductions in resting metabolic rate (RMR) beyond that which can be accounted for by weight loss alone. Since RMR is a primary contributor to the daily energy budget ^9^, this represents a significant barrier to long-term weight loss. It is notable that exercise alone is only marginally effective as a therapy for weight loss ^10– 12^. This is likely due to both metabolic and behavioral obstacles. Exercise training fails to increase RMR in obese individuals with diabetes ^13^, and this is potentially due to increased metabolic efficiency ^14^. Exercise training has had mixed results in eliciting weight loss in both rodents ^15^ and humans ^10^ and is explained in part by increased food intake. In addition to RMR, “non-exercise activity thermogenesis” (NEAT) is a major contributor to energy expenditure in mice and humans ^16^. Mice given access to a running wheel increase their physical activity and energy expenditure over a four-week period, but the metabolic cost of activity progressively decreases concurrently with decreased NEAT ^17^. This is significant because fat gain with overnutrition in humans is positively correlated with an increase in NEAT ^18^. Whether the bidirectional modulation of NEAT based on whole-body energy balance is modifiable therapeutically remains to be seen but may be a viable strategy for combating obesity. Complicating therapeutic strategies further is a growing body of literature demonstrating that the metabolic adaptations that occur with weight loss predispose an individual to accelerated weight regain and increased adiposity upon cessation of a supervised diet or exercise regimen or both ^19^. Notably, recent work suggests that glucocorticoid antagonism mitigates the weight regain and IR that occur following cessation of voluntary exercise in rats ^20^. A better understanding of how humans resist weight loss, even in the setting of obesity, is critically important in that it may reveal novel therapeutic strategies for treating obesity and IR.

## Mitochondrial energetics and the pathogenesis of insulin resistance

As the demand-driven terminus of oxidative metabolism, mitochondria are intricately involved in the maintenance of energy balance, and several recent reviews have highlighted the importance of mitochondrial energetics to the etiology of IR ^21– 23^. At the level of the mitochondrion, energy balance is established by a dynamic rate of carbon flux through the tricarboxylic acid (TCA) cycle that supports ATP production via oxidative phosphorylation. In the setting of overnutrition, there is a supply/demand mismatch that results in excess anaplerotic flux of carbon from fatty acids entering the TCA cycle relative to the ATP demand leading to IR ^24^. Excessive anaplerotic flux creates a mitochondrial “carbon stress” that has been well documented in both skeletal muscle (SkM) and liver. This carbon stress promotes IR through incompletely defined mechanisms that likely involve post-translational protein modifications that alter insulin signaling or protein trafficking (that is, GLUT4 translocation). The teleological explanation for limiting SkM glucose uptake in the face of excess dietary lipids may be that SkM is unable to efficiently convert excess intracellular glucose to an inert metabolite (that is, fatty acids).

In the liver, greater fatty acid availability accelerates anaplerotic flux contributing to IR that correlates with the severity of non-alcoholic fatty liver disease (NAFLD) in humans ^25^. This appears to be linked, at least in part, to incomplete β-oxidation in the setting of overnutrition ^26^. This hypothesis is supported by findings that acyl-carnitine, the carbon chain intermediate of β-oxidation, is increased in human plasma ^27^ as well as rodent SkM ^26^. Free carnitine in SkM is also reduced in the setting of obesity or high-fat feeding or both ^28^, suggesting a reduced capacity to handle excess fatty acids. Collectively, excess dietary fatty acids entering metabolically active tissues overload the mitochondria, leading to IR. A teleological explanation for why mitochondria induce IR may be to mitigate oxidative damage induced by overnutrition ^29^.

Mitochondria can also engage in cataplerosis, which is removal of carbons from the TCA cycle. In SkM, one proposed role for cataplerosis is as a buffering system to avoid mitochondrial carbon excess that can lead to increased reactive oxygen species (ROS) production during overnutrition ^24^. SkM cataplerosis occurs in large part via carnitine acetyltransferase (CrAT), an enzyme that is responsible for exporting acetyl and acyl groups bound to carnitine from the mitochondrial matrix into the cytosol. Mice with SkM-specific deletion of CrAT have impaired glucose tolerance and increased oxidative stress ^30^, illustrating a need for mitochondrial carbon efflux (that is, cataplerosis) to preserve SkM metabolic homeostasis in the setting of overnutrition. In the liver, cataplerosis is essential for the production of both glucose (gluconeogenesis) and ketones (ketogenesis). Predominantly expressed in gluconeogenic organs (liver and kidney), phosphoenolpyruvate carboxykinase (PEPCK) converts oxaloacetate to pyruvate and is a key enzyme for gluconeogenesis. Loss of PEPCK in mice reduces hyperglycemia in leptin receptor–deficient (db/db) diabetic mice ^31^. Similarly, ketogenesis exerts partial protection against high-fat diet (60% calories from fat)–induced hyperglycemia and fatty liver, primary complications linked to obesity and overnutrition ^32^. Notably, however, mice fed a ketogenic diet (more than 90% calories from fat) are lean and hypoinsulinemic but also display fatty liver ^33, 34^. This may be due to the impaired liver mitochondrial respiratory capacity observed in mice fed a short-term (14 days) ketogenic diet ^35^. Strategies to increase cataplerosis in a tissue- and product-specific fashion could yield valuable strategies for preserving glucose homeostasis and insulin sensitivity but should be considered in the context of also preventing the development of fatty liver.

How does mitochondrial carbon excess promote IR? Carbon turnover that exceeds metabolic demand leads to accumulation of reducing equivalents (NADH and FADH _2_) that exert greater “reducing pressure” (that is, more electrons) on the electron transport system ^36^. This buildup of reducing equivalents in the matrix and electrons within the electron transport system promotes the formation of ROS that modulate a wide variety of normal and pathophysiological cellular processes ^37^. For example, acute or chronic high-fat feeding increases mitochondrial ROS production that has been shown in some ^29, 38– 40^, but not all ^41^, reports to be causal for the development of IR. Notably, fatty acids can also “uncouple” oxidative phosphorylation ^42^, raising the possibility that mitochondrial oxidative efficiency may be an additional mechanism to manage carbon excess in obesity. Targeting this mechanism may be feasible in light of recent work demonstrating that mitochondrial oxidative efficiency is a dynamic process that is acutely sensitive to energetic demand ^43^. Historically, the use of mitochondrial uncouplers as therapeutic agents has been met with skepticism following a string of deaths linked to the protonophore 2-dinitrophenol in the 1930s. However, recent efforts have provided new lead compounds that may be promising in the treatment of obesity ^44– 46^. While mitochondria-targeted therapies are being studied intensively and hold great promise, an alternative approach may be to address downstream effectors of mitochondrial oxidants. The downstream processes affected by mitochondrial oxidants are incompletely defined but include inflammation and expansion of the ECM. The remainder of this article will be spent discussing these processes in the context of their individual, and collective, contributions to the etiology of IR.

## Inflammation and extracellular matrix expansion in the etiology of insulin resistance

Low-grade metabolically driven “meta-inflammation” ^47^ contributes to IR in obesity ^48^. There are numerous intersecting mechanisms linking inflammation and ROS ^49^, including a critical role for the innate immune system that is coupled to macrophage infiltration ^50, 51^. Macrophages recruited with chronic overnutrition are pro-inflammatory (M1; CD11b ^+^) and secrete tumor necrosis factor alpha (TNFα) that has been shown to contribute to IR in adipose, SkM, and liver ^52– 54^. M1 macrophages also play a critical role in wound healing. It has been observed that the meta-inflammatory response to obesity that includes M1 macrophage infiltration is responsible for the accumulation of ECM proteins in insulin-sensitive tissues ^55^. The evidence linking these processes in adipose, SkM, and liver is outlined below.

Adipose tissue function is reliant upon, and in certain situations compromised by, the ECM surrounding adipocytes ^56^. Healthy adipose tissue expansion involves a balance between enzymatic degradation and subsequent synthesis of ECM proteins ^57^. Pathogenic obesity in humans is characterized by adipose tissue fibrosis due to excessive ECM deposition and reduced ECM degradation that is associated with IR ^58– 61^. Paradoxically, recent work by Muir *et al*. ^62^ showed that diabetics have reduced adipose tissue fibrosis and greater adipose tissue hypertrophy. Genetically obese ( *ob/ob*) mice have increased expression of genes encoding collagens ^63^ that is exacerbated by high-fat feeding ^64^. Genetic loss of the adipose tissue–abundant collagen VI in mice mitigates adipocyte inflammation, diet-induced obesity (DIO), and glucose intolerance while permitting greater adipocyte hypertrophy ^63^. Beyond collagen, various other ECM components—including osteopontin ^65, 66^, hyaluronan ^67^, thrombospondins ^68, 69^, and microfibril-associated glycoprotein 1 (MAGP1) ^70^—accumulate in adipose tissue with obesity and contribute to IR. Adipose tissue ECM expansion is attenuated by the anti-diabetic drug metformin ^71^, a drug that is also known to reduce mitochondrial ROS production ^39^. Whether metformin improves metabolic health by mitigating mitochondrial ROS production or ECM accumulation or both remains to be addressed directly.

Obesity induces SkM ECM expansion ^55, 72, 73^ that would be expected to increase the resistance to glucose delivery, an essential controller of glucose uptake ^74^. Even short-term (28 days) high-fat feeding ^75^ is sufficient to induce SkM ECM expansion. This appears to be reversible as SkM collagen accumulation is ameliorated in obese mice following exercise training ^73^ and preventable in mice with genetic enhancement of SkM mitochondrial ROS scavenging ^55^. A genetic knockout of matrix metalloprotease-9 (MMP-9), a key ECM-degrading enzyme, in obese mice causes increased collagen and a further deterioration of SkM insulin action ^76^. Treatment with pegylated hyaluronidase causes degradation of hyaluronan and rescues IR in obese mice ^77^. These studies demonstrate a direct link between ECM accumulation and insulin action in SkM.

In the setting of obesity, circulating lipids are incompletely sequestered in adipose tissue and consequently accumulate in SkM and liver and lead to IR. NAFLD is a primary risk factor for the development of IR and diabetes via liver fibrosis ^78, 79^. Mice fed a high-fat high-fructose diet exhibit liver fibrosis that accompanies lipid accumulation and IR ^80, 81^. The extent and scope to which overnutrition alters liver ECM are not completely known, highlighting a need for future studies.

ECM accumulation is recognized as a structural barrier between cells and the vascular space that restricts molecular transport ^82^. More recently, a body of evidence has emerged indicating that cellular changes that accompany ECM accumulation are receptor-mediated. As such, the ECM is a biomolecular “motherboard” that determines the physical and metabolic properties of the tissue and the cells that they envelope. A greater understanding of how the ECM changes in obesity and the contribution of individual ECM proteins will be necessary in defining extracellular processes impacting metabolic health.

## Extracellular matrix expansion and integrins in the setting of obesity

**Figure 2.**
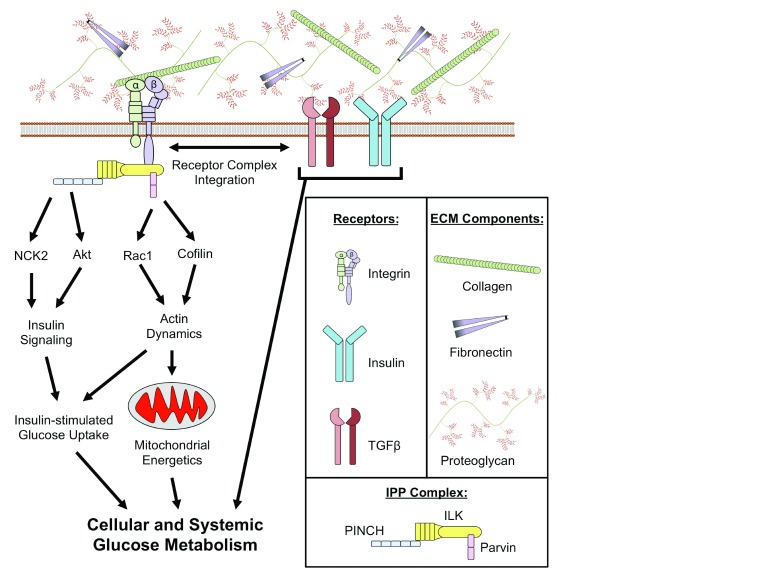
Putative mechanisms for the role of integrins in the development of insulin resistance. Extracellular matrix (ECM) proteins are ligands for integrins, a family of cell surface receptors. Integrins are linked to the regulation of glucose metabolism through numerous mechanisms. Integrins can co-localize with transforming growth factor beta (TGFβ) and insulin receptors that are key regulators of glucose uptake into tissues. Integrins are also involved in intracellular signaling through the integrin-linked kinase (ILK)/PINCH/Parvin (IPP) complex. PINCH is characterized as a modulator of kinase signaling pathways as it regulates Nck2 and Akt, requisite proteins for insulin signaling. Parvin is involved in the regulation of cytoskeletal dynamics that permit remodeling and translocation of mitochondria and various intracellular proteins (that is, glucose transporters). The integration of integrins with regulatory nodes for glucose metabolism highlights the potential significance of ECM-integrin signaling in the etiology of insulin resistance.

Integrins are a class of receptors that bind ECM proteins and have numerous overlapping functions, including cell adhesion, mechanotransduction, and differentiation ^83, 84^. ECM receptors are involved in a myriad of receptor signaling events through physical and functional interactions with growth factor receptors, including the insulin receptor ^85^. In this way, ECM receptors orchestrate dynamic and specific signaling responses to diverse physiological and pathophysiological conditions. Integrins functionally link ECM changes to a multitude of conditions, including IR ^86^ (summarized in Figure 2).

 Integrins are heterodimers consisting of α and β subunits with varying ligand specificities and expression in different tissues. Differentiated insulin-sensitive cells from SkM, adipose tissue, and liver express a variety of α subunit isoforms but express only a single β integrin isoform (β1) ^87– 89^. Whole-body loss of the integrin α1 subunit, a pro-fibrotic integrin receptor subunit that exclusively binds to β1, fails to protect against diet-induced SkM IR in mice; however, loss of the anti-fibrotic α2 isoform that also binds to β1 is protective ^55^. It is interesting to note that combined SkM and myocardial loss of the integrin β1 subunit results in IR in lean mice ^90^. Integrin-linked kinase (ILK) is a protein that physically associates with the intracellular tail of the β integrin subunit ^90^. In contrast to the IR caused by knockout of the integrin β1 subunit in both SkM and myocardium of lean mice ^90^, SkM-specific loss of ILK (mILK-KO mice) results in improved SkM insulin action in DIO mice ^91^. Liver-specific deletion of ILK also protects against IR in DIO mice ^92^. Whether adipocyte ILK deletion has effects on nutrient metabolism remains to be determined.

Despite its name, ILK lacks a functional kinase domain but rather functions as a scaffold for at least 26 high-fidelity binding partners ^93^. Most notable among these binding proteins are PINCH and parvin, which, together with ILK, form an ILK/PINCH/Parvin (IPP) complex. PINCH consists of two isoforms (PINCH1 and PINCH2) that have both distinct and overlapping cellular functions ^94^. In the context of glucose homeostasis, PINCH can bind to Nck2, which in turn interacts with insulin receptor substrate-1 (IRS-1) ^95^, a requisite for insulin signaling. Nck2 is highly expressed in epididymal adipose tissue and its genetic deletion in mice causes IR and increased lipolysis ^96^. PINCH has also been implicated in the phosphorylation of Akt via interactions with ILK ^97^. Three ubiquitously expressed isoforms of parvin exist (α, β, and γ). α- and β-parvin both can bind directly to f-actin and in this way regulate cytoskeletal dynamics ^98^. Parvin-mediated regulation of actin cytoskeletal dynamics is thought to occur, at least in part, via interactions between parvin and the Rho GTPase Rac1 ^99, 100^ and actin depolymerizing factor protein cofilin ^101^. Rac1 is required for insulin-stimulated glucose uptake and is impaired during IR ^102^, representing a potential link between integrins and insulin action. A potential role for γ-parvin in the context of insulin action has not been elucidated. Rac1 and cofilin are also involved in the regulation of numerous mitochondrial processes, including fission ^103^, apoptosis ^104^, and translocation ^105^, demonstrating a link between integrins and the regulation of oxidative metabolism. Whether integrins and the IPP complex directly regulate Rac1 or cofilin in obesity is not yet known, nor is it known what role the IPP complex may play in obesity through its other binding partners. A recent report shows that focal adhesion kinase (FAK), an alternative downstream target of integrin activation, can modulate insulin sensitivity through regulation of adipocyte survival ^106^. In light of the complexities of the ECM, integrins, and intracellular signaling pathways, much remains to be learned about ECM-integrin interactions in IR.

## Summary and future directions

The etiology of IR involves both cell-intrinsic regulation of nutrient metabolism and integrated systems pathophysiology. The established paradigm of meta-inflammation coupled with the emerging concept of meta-fibrosis illustrates the complex nature of IR; however, several major questions remain to be addressed. For example, the composition and organization of the ECM must be elucidated so that the contribution of individual proteins or complexes or both can be mechanistically understood. Additionally, the role of downstream intracellular substrates of integrin signaling must be defined in the context of IR and cellular metabolism. The complex nature and broad importance of ECM/integrin function will be better understood through interdisciplinary studies that draw expertise from numerous fields (such as mechanobiology, biophysics, endocrinology, and molecular metabolism). It is anticipated that future studies will provide a more complete understanding of how the ECM functions as a biophysical regulator of whole-body function and support the development of novel therapeutics aimed at treating IR by mitigating meta-fibrosis.
